# Human P301L-Mutant Tau Expression in Mouse Entorhinal-Hippocampal Network Causes Tau Aggregation and Presynaptic Pathology but No Cognitive Deficits

**DOI:** 10.1371/journal.pone.0045881

**Published:** 2012-09-24

**Authors:** Julie A. Harris, Akihiko Koyama, Sumihiro Maeda, Kaitlyn Ho, Nino Devidze, Dena B. Dubal, Gui-Qiu Yu, Eliezer Masliah, Lennart Mucke

**Affiliations:** 1 Gladstone Institute of Neurological Disease, San Francisco, California, United States of America; 2 Department of Neurology, University of California San Francisco, San Francisco, California, United States of America; 3 Department of Neurosciences, University of California San Diego, La Jolla, California, United States of America; 4 Department of Pathology, University of California San Diego, La Jolla, California, United States of America; Boston University School of Medicine, United States of America

## Abstract

Accumulation of hyperphosphorylated tau in the entorhinal cortex (EC) is one of the earliest pathological hallmarks in patients with Alzheimer’s disease (AD). It can occur before significant Aβ deposition and appears to “spread” into anatomically connected brain regions. To determine whether this early-stage pathology is sufficient to cause disease progression and cognitive decline in experimental models, we overexpressed mutant human tau (hTauP301L) predominantly in layer II/III neurons of the mouse EC. Cognitive functions remained normal in mice at 4, 8, 12 and 16 months of age, despite early and extensive tau accumulation in the EC. Perforant path (PP) axon terminals within the dentate gyrus (DG) contained abnormal conformations of tau even in young EC-hTau mice, and phosphorylated tau increased with age in both the EC and PP. In old mice, ultrastructural alterations in presynaptic terminals were observed at PP-to-granule cell synapses. Phosphorylated tau was more abundant in presynaptic than postsynaptic elements. Human and pathological tau was also detected within hippocampal neurons of this mouse model. Thus, hTauP301L accumulation predominantly in the EC and related presynaptic pathology in hippocampal circuits was not sufficient to cause robust cognitive deficits within the age range analyzed here.

## Introduction

Alzheimer’s disease (AD) continues to be a devastating and mostly untreatable disease [1]. Research has focused on two key proteins, Aβ and tau, identified as the central components of the main pathological hallmarks of AD. Whether Aβ and tau play causal roles and how they interact in the majority of AD cases is uncertain. Mutations in genes affecting Aβ production cause rare early-onset forms of AD [2], whereas mutations in the human tau gene (*MAPT*) do not appear to cause AD. Known *MAPT* mutations lead to frontotemporal dementia and parkinsonism linked to chromosome 17 (FTDP-17) [3], and other forms of frontotemporal lobar degeneration [4]. Widespread neuronal overexpression of *MAPT* bearing the FTDP-17-linked P301L mutation in transgenic mice causes motor impairments, as well as cognitive decline and tau pathologies reminiscent of AD, but in the absence of Aβ accumulation [5], [6].

Before clinical symptoms emerge in AD, pathological forms of tau are confined to certain brain regions [7]–[9]. Cortical involvement begins in entorhinal (EC) and transentorhinal cortex (Braak stage I), followed by the hippocampal CA1 region (Braak stage II). These early Braak stages can occur without Aβ deposits [10], [11]. The EC is also arguably the earliest region with neuronal loss, hypometabolism, and atrophy in mild cognitive impairment or early AD patients [12], [13]. Normal EC function is essential to many types of memory [14], [15], including spatial memory, which is impaired in AD patients [16], [17]. In later Braak stages associated with clinical impairments, tau pathology increases in all hippocampal subregions and then neocortical regions. “Spread” of tau pathology appears to occur between synaptically connected regions, but the mechanisms of propagation between brain areas are unknown. One possibility is that aggregated tau could move between cells [18], [19]; acting in a prion-like fashion to seed further accumulations of tau [20]–[22].

Transgenic mice with widespread overexpression of human tau are not suited to address the question of whether primary tau pathology within EC causes cognitive problems or disease progression through circuits. We generated transgenic mice expressing human P301L-mutant tau (hTau) in brain areas affected in early AD (Braak stages I/II). We investigated whether hTau can initiate disease progression in the absence of human Aβ and analyzed the effect of hTau expression at synapses of perforant path (PP) axons in the dentate gyrus (DG). Overexpression of hTau in the EC caused substantial hyperphosphorylation and abnormal conformations of tau locally and in PP and DG granule cells (GC) of young mice, which increased with age. However, even with extensive tau pathology in this network, mice showed no cognitive deficits up to 16 months of age, which is in contrast to transgenic mice expressing mutant APP/Aβ in a similar topographical pattern [23].

## Materials and Methods

### Animals

Tet-hTauP301L responder transgenic mice (FVB/NCr strain) [24] were provided by Dr. Jada Lewis. Transactivator neuropsin-tTA transgenic mice enabling spatially restricted expression predominantly in the EC and parahippocampal regions (C57BL6 strain) [25] were provided by Dr. Mark Mayford. Heterozygous mice from each line were bred to generate mice of four genotypes: neuropsin-tTA/tet-hTau doubly transgenic (EC-hTau) mice, neuropsin-tTA or tet-hTau singly transgenic mice, and NTG controls. C57/FVB F1 mice were used for all studies. Doxycycline was not administered so as to achieve constitutive expression of the tet-hTau transgene in EC-hTau mice throughout life. Experimenters were blinded to genotype for all experiments. The Institutional Animal Care and Use Committee of the University of California, San Francisco approved all experiments.

### Behavioral Tests

Cohorts of male and female mice were behaviorally evaluated at 4, 8, and 12 months of age. All four genotypes were tested: NTG (n = 9 female, 14 male), neuropsin-tTA (n = 13 female, 9 male), tet-hTau (n = 10 female, 15 male) and EC-hTau (n = 14 female, 8 male). Male mice were also tested at 16 months of age. Because no statistical differences were observed between males and females, data from both sexes were combined at 4, 8 and 12 months. The same mice were tested at 4, 8, 12 and 16 months in the Morris water maze. In addition, they were assessed for novel object recognition (4, 12, 16 months), contextual fear conditioning (12 months) and novel place recognition (16 months). An independent cohort of mice was also tested in the Morris Water Maze at 8 months of age only (n = 11–12/genotype split evenly between male and females).

### Morris Water Maze

Mice were trained to locate a hidden platform in the Morris water maze (MWM) as described [23], [26]. Latencies to find the platform, distances traveled, swim paths, swim speeds, percent time spent in each pool quadrant, and platform crossings were all recorded for subsequent analyses. Behavior was recorded with a video tracking system (Noldus).

### Novel Object Recognition and Novel Place Recognition

Novelty recognition memory was tested for objects and location. Mice were transferred to the testing room and given at least 1 hr to acclimate. On days 1 and 2, mice were habituated to the testing arena (white 40×40 cm square plastic chambers lit by red light) for 15 min. On the third day, mice were placed in the same arena and presented with two identical objects evenly spaced, which they could explore for 10 min. For novel object recognition testing, mice were presented with two objects 4 hours later: one was an exact replica of the one used in the training phase and the other was a novel, unfamiliar object with different shape and texture. Positions of both objects were counter-balanced across the experiment. For novel place recognition testing, mice were presented with the same two objects 3 hours after the initial exploration: one was in the exact location as in the training phase, whereas the other was in a novel location. They were allowed to explore the objects for 10 min. Arenas as well as objects were cleaned with 70% ethanol between each mouse. Frequency of visits and the time spent exploring each object was recorded for subsequent data analysis. Behavior was recorded onto a digital camcorder and the data was analyzed manually using a stopwatch.

### Contextual Fear Conditioning

Mice were tested for their ability to remember the context in which they received a foot shock. All mice were initially acclimated to the testing chamber for 5 min 24 hours before training. During training, mice were placed into the cage for 6 min. First, baseline freezing activity was recorded for 3 min. A series of three 2-sec 0.4-mA foot shocks was then given in 60-sec intervals. Twenty-four hours later, mice were returned to the chamber for 5 min without receiving shocks, and the time they spent freezing was recorded for analysis.

### Immunohistochemistry and Histology

Brain tissue was obtained from EC-hTau mice at 4 months (n = 3 females, 4 males), 8 months (n = 5 females, 5 males), 12 months (n = 1 female, 7 males), and 16 months (n = 5 females, 8 males) of age and prepared for immunostaining as described [23], [26]. In each of the four age groups, sections from 1 NTG, 1 neuropsin-tTA, and 4 tet-hTau mice were included as controls. Primary mouse antibodies against tau included MC1, CP13, and PHF1 (1∶1000, 1∶2000, 1∶1000, gifts from Dr. Peter Davies, Albert Einstein College of Medicine), HT7 (1∶1000; Thermo Scientific, Rockford, IL), and AT8 (1∶250; Thermo Scientific). Binding of these antibodies was detected with biotinylated donkey anti-mouse (1∶1000; Jackson Immunoresearch), followed by incubation with avidin-biotin complex (Vector) and visualization with DAB (Sigma). Synaptic and subcellular markers were detected with mouse anti-synaptophysin (1∶1000; Boehringer Mannheim), rabbit anti-synapsin I (1∶500; Millipore, Billerica, MA**)**, and mouse anti-MAP2 (1∶100; Millipore). Anti-synaptophysin and anti-MAP2 antibodies were detected using Tyramide Red (Perkin Elmer, Waltham, MA). Forty µm thick vibratome sections were mounted onto coated glass slides and stained with the Gallyas silver protocol as described [27]. Briefly, sections were placed in 5% periodic acid followed by alkaline silver iodide solution and developer solution. After washing with acetic acid and water, they were placed in 0.1% gold chloride, followed by sodium thiosulphate solution, washed and counterstained in 0.1% nuclear fast red.

Synaptophysin and synapsin immunoreactivities were quantified in 16-month-old male NTG (n = 8) and EC-hTau (n = 8) mice essentially as described [28]. Briefly, synaptophysin-immunostained sections were imaged with a laser scanning confocal microscope, and percent area covered by synaptophysin-immunoreactive terminals was determined with Image-Pro Plus (Media Cybernetics, Bethesda, MD). DAB-reacted sections labeled for synapsin were imaged on an Olympus digital microscope; optical density measurements were performed with ImageJ. Sections from 16-month-old EC-hTau and NTG mice were colabeled with PHF1 and antibodies against synaptophysin or MAP-2 followed by detection with fluorescent secondary antibodies. The percent overlap (yellow) between red and green signals was estimated with Image-Pro Plus software on digital images obtained from a laser scanning confocal microscope.

### Electron Microscopy

Vibratome sections from 16-month-old EC-hTau and NTG mice were postfixed in 1% glutaraldehyde, treated with osmium tetraoxide, embedded in epon araldite and sectioned with an ultramicrotome (Leica, Germany). Grids were analyzed with a Zeiss OM 10 electron microscope as described [29]. For immunogold labeling, sections were mounted in nickel grids, etched and incubated with PHF1. Secondary antibodies tagged with 10-nm Aurion ImmunoGold particles (1∶50, Electron Microscopy Sciences, Fort Washington, PA) with silver enhancement were used for detection. A total of 250 synapses were analyzed per mouse. Synapses were randomly acquired from 3 grids and electron micrographs were obtained at a magnification of 25,000X. Electron micrographs were digitized and analyzed with NIH Image 1.43 software to determine the number of gold particles in pre- versus post-synaptic sites [29].

### Immunoblotting

DG and EC samples were microdissected from NTG and EC-hTau brain tissues and homogenized in TBS containing Phosphatase Inhibitor Cocktails I and II (Sigma-Aldrich, St. Louis, MO) and protease inhibitors (Roche, Indianapolis, IN). The homogenate was spun at 13,000×g for 10 min and the supernatant was used as the total fraction. The total fraction was spun at 150,000×g for 15 min. The precipitate was dissolved in 2% sarcosyl and incubated for 2 hrs, then spun at 150,000×g for 15 min. The final precipitate was used as the insoluble fraction. The loading amount of the total fraction was adjusted by the total amount of protein. The loading amount of insoluble tau was adjusted by the total amount of protein in the starting material [30]. Proteins were transferred to nitrocellulose membranes with the iBlot system (Invitrogen, Carlsbad, CA). The membranes were probed with antibodies against tau (Tau5 1:5000; Millipore, Billerica, MA) and GAPDH (1∶5000; Novus, Littleton, CO), followed by IRdye 680CW goat anti-rabbit IgG and IRdye 800CW goat anti-mouse IgG (Licor, Lincoln, NB). The membranes were analyzed by Odyssey infrared imaging scanner (Licor, Lincoln, NB). Hippocampal samples from rTg4510 mice, which express high levels of the tet-hTauP301L transgene in the hippocampus [30], were used as controls.

### Statistical Analyses

Experimenters were blinded to the genotype of mice in all studies. Latencies in the Morris water maze were analyzed with mixed-model ANOVAs [31]. In this approach, a random effect is used to control for the repeated measures from a particular mouse. Genotype and day of trial are fixed effects and all two- and three-way interactions between these effects were explored. Sex was also initially included as a fixed effect, but was removed due to a lack of significance. Probe trials and all other behavioral tasks (novel object, novel place, contextual fear conditioning) were analyzed with ANOVA and post hoc tests as indicated in figure legends. Statistical power curves were calculated using the sample sizes and observed variability for each dataset from the water maze, novel object and novel place tests across ages. The values at which 80% power detection, or a type II error rate of beta = 0.2, would be achieved are reported in the text. Differences between means for histological quantifications were analyzed by unpaired two-tailed t-tests or ANOVA with post hoc tests as appropriate. p<0.05 was considered significant.

## Results

### Expression of P301L-mutant hTau in Neuropsin-tTA/tet-hTau Doubly Transgenic (EC-hTau) Mice

**Figure 1:**
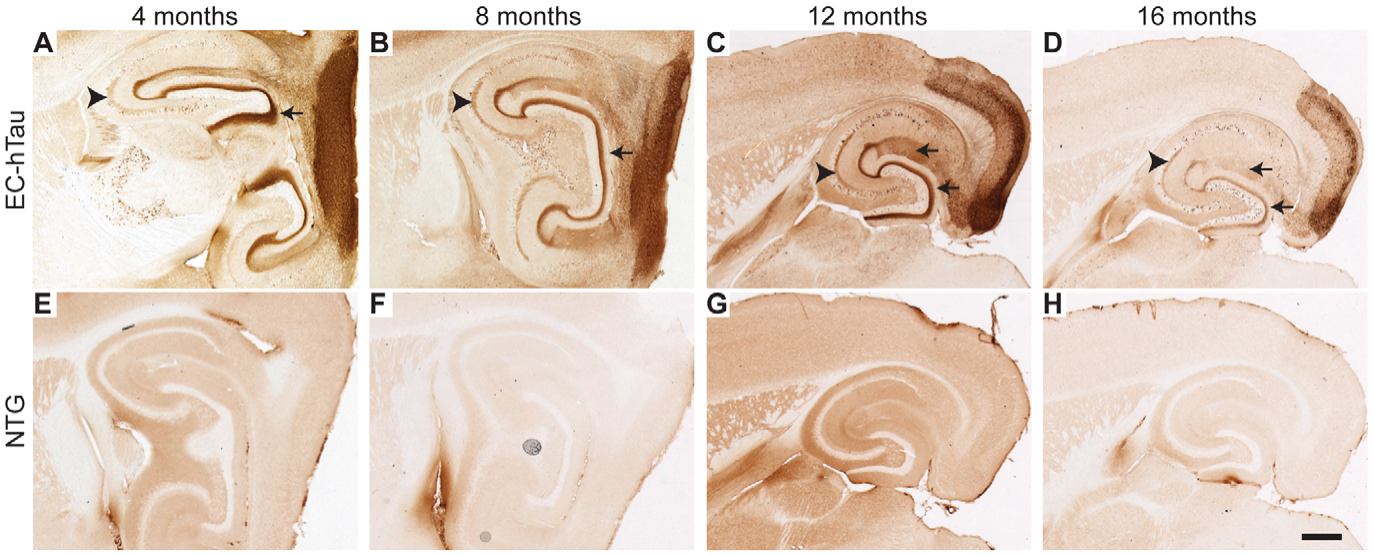
Spatially restricted expression of P301L-mutant hTau in EC-hTau mice. Representative images of sagittal (A, B, E, F) and horizontal (C, D, G, H) brain sections from 4-, 8-, 12-, and 16-month-old EC-hTau mice (top) and NTG controls (bottom) immunostained for hTau with the anti-hTau antibody HT7 (A–D). In EC-hTau mice, hTau expression was observed primarily in cell bodies and neuropil of the EC and in PP terminals in the hippocampus (arrows). Mossy fiber axons of DG GC also stained for hTau (arrowheads). From 8 months onward, there was labeling of scattered cells in the DG, CA3 and CA1 regions in the hippocampus. (E–H) Only nonspecific background staining was observed in NTG mice. Scale bar (H) = 500 µm.

We bred neuropsin-tTA transgenic mice [25] with tet-hTauP301L mice [6] to generate mice with P301L-mutant hTau expression restricted primarily to the entorhinal cortex. Neuropsin-tTA/tet-hTau doubly transgenic offspring (EC-hTau mice) were raised free of doxycycline to keep hTau expression “on” at all times. Location of the hTau transgene was examined by immunostaining of brain sections from 4–16-month-old mice with the HT7 antibody, which is specific to hTau. As expected based on other studies with the neuropsin-tTA line [23], [25], EC-hTau mice had high levels of hTau in EC neurons and in PP terminals within the hippocampus (Figure 1A–D). There was no obvious change in expression level of hTau in the EC with increasing age (Figure 1A–D). No hTau was detected in the EC of NTG mice at any age (Figure 1E–H) or in mice singly transgenic for neuropsin-tTA (data not shown) or tet-hTau (Figure S1) by immunostaining.

### Expression of P301L-mutant hTau in the EC and Hippocampus is not Sufficient to Cause Learning and Memory Deficits

**Figure 2:**
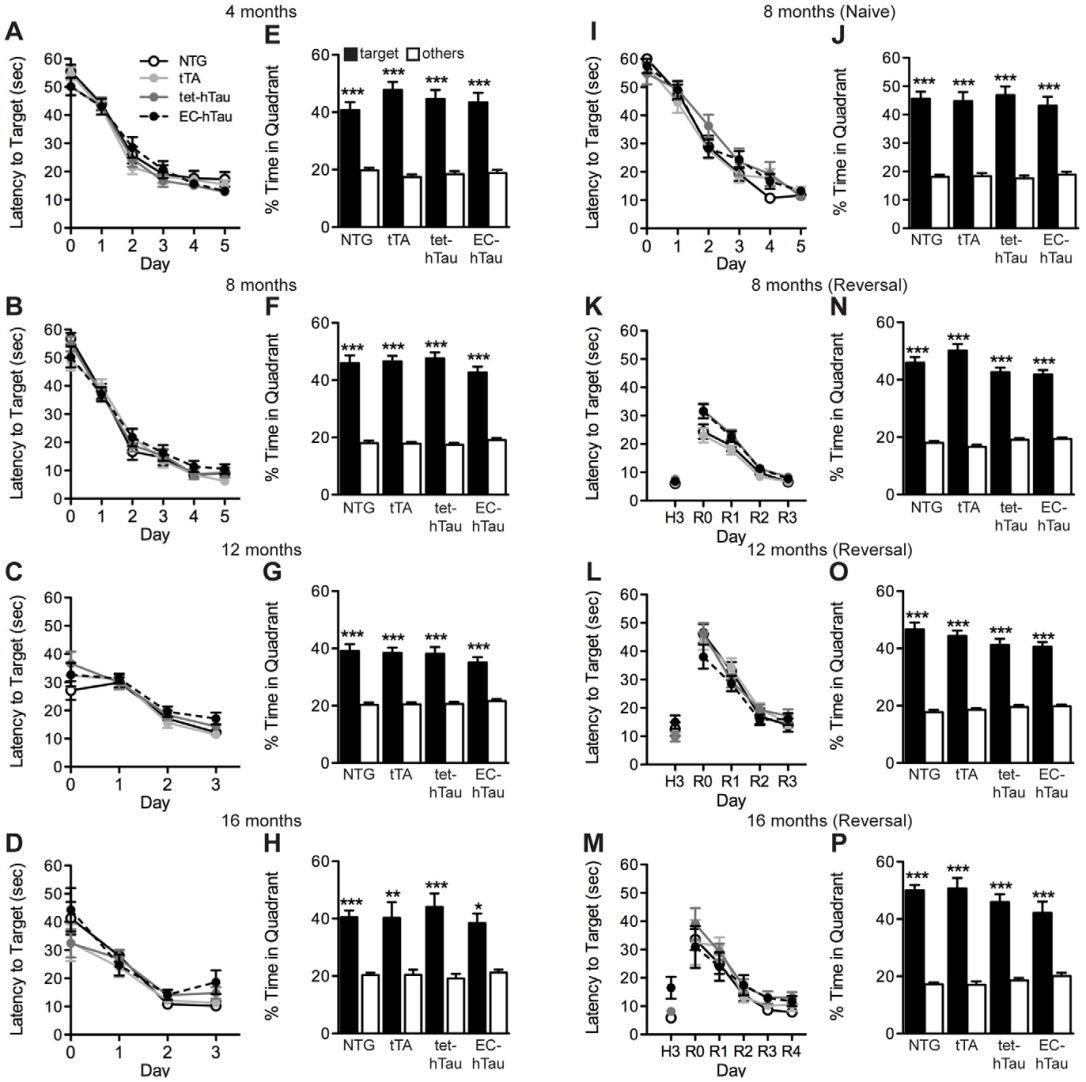
EC-hTau mice do not have deficits in the Morris water maze (MWM). (A–H) Mice from each of the four genotypes were tested at 4, 8, 12 and 16 months of age in the MWM. (A–D) Learning curves. (E–H) Twenty-four hours after the last training session, spatial memory was tested in a probe trial. (I, J) An independent cohort of 8-month-old behaviorally naïve EC-hTau mice also showed normal learning (I) and memory retention (J). (K–P) EC-hTau mice also displayed normal reversal-learning in the MWM. (K–M) After the initial training to find a hidden platform, the platform was moved to a new location in the opposite pool quadrant. Mice were then trained to navigate to this new location for 3 days. (N–P) Twenty-four hours after the last reversal training session, spatial memory was tested in a probe trial. *p<0.05, **p<0.005, ***p<0.0005 vs. the average percent time spent in non-target quadrants (Tukey test). Values are mean ± SEM.

Widespread overexpression of mutant hTau throughout the forebrain and hippocampus causes learning and memory impairments [6], [32]–[34]. To determine whether overexpressing hTau at high levels within the EC and parahippocampal regions and low levels in the hippocampus causes similar cognitive deficits, we tested groups of EC-hTau and control mice repeatedly in the Morris water maze at 4, 8, 12, and 16 months of age (Figure 2). EC-hTau mice learned to navigate to the hidden platform as well as the control groups at all ages examined, as reflected in the latencies to reach the hidden platform (Figure 2A–D). Distances to reach the target platform were also recorded and yielded similar results (data not shown). To assess the validity of these findings, we calculated statistical power curves for latency and distance measures at each age based on our sample sizes (data not shown). This analysis indicated that our study was well-powered (type II error rate of beta <0.2) to detect a significant change in the average latency in the EC-hTau mice by 9.35 sec (4 months), 9.4 sec (8 months), 6.2 sec (12 months) and 8.6 sec (16 months) with a type I error rate of alpha = 0.05.

Memory for the platform location was tested in a probe trial (platform removed) 24 hours after the last training session. EC-hTau mice again performed at the same level as the control groups in this test of spatial memory (Figure 2E–H). All mice spent significantly more time searching in the target quadrant, where the platform had been located, than in the other quadrants.

We also assessed the performance of an independent cohort of mice to make sure that subtle learning and memory deficits were not being obscured by repeated assessment in the same test, which might help impaired mice perform the task better. Behaviorally naïve EC-hTau mice tested only at 8 months of age were also indistinguishable from control groups in the Morris water maze test (Figure 2I, J). We previously showed that overexpression of mutant APP in the EC causes deficits in a reversal learning variation of this test [23]. We therefore tested the EC-hTau mice in this reversal paradigm, which requires mice to abandon a previously learned platform location and to learn to navigate to a new location. Unlike EC-APP mice, EC-hTau mice had no difficulty with this task at 8, 12 or 16 months of age (Figure 2K–P). Based on sample sizes and calculation of statistical power curves (not shown), our reversal test would have been sensitive enough (type II error rate beta <0.2) to detect a significant change in latency of 4.2 sec (8 months), 10.3 sec (12 months), and 10.2 sec (16 months) in the EC-hTau group with a type I error rate of alpha = 0.05.

**Figure 3:**
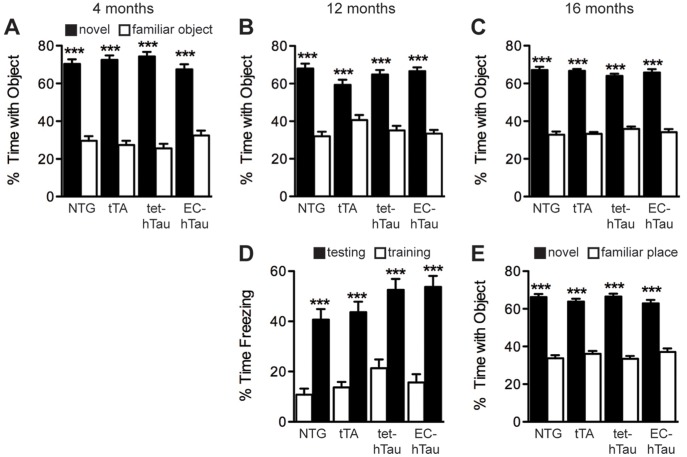
EC-hTau mice do not have deficits in other tests of learning and memory. (A–C) Mice of the four genotypes were tested in a novel object recognition task at 4 (A), 12 (B), and 16 (C) months of age. At all ages, all groups of mice spent significantly more time with the novel object, indicating memory of the familiar object. (D) At 12 months, mice were tested in a fear-conditioning task. Mice from all genotypes spent significantly more time freezing in the test session 24 hours after training. (E) At 16 months, mice were tested in a novel place recognition task. Again, all genotypes spent significantly more time with the object in the novel location, indicating memory for the familiar place. ***p<0.0005 vs. empty bars (Tukey test). Values are mean ± SEM.

We also assessed learning and memory in other cognitive tasks that have revealed deficits in APP transgenic mouse lines [26], [35]. At 4, 12, and 16 months, recognition memory was tested in a novel object paradigm (Figure 3A–C). Like the control groups, EC-hTau mice spent significantly more time exploring a novel object when presented with both the novel object and a familiar object, indicating memory of the familiar object. There was also no difference between EC-hTau and NTG mice in time spent exploring the new objects (two-sample t-test) at any age. Power analyses indicated that, depending on age, a change of 9.6–11.5% would have been detectable as a significant effect of EC-hTau genotype with the current sample sizes. At 12 months, mice were tested in a contextual fear paradigm. All groups of mice, including the EC-hTau mice, formed strong memories of receiving a foot shock, as shown by increased time spent freezing in the arena where a shock was received 24 hours ago (Figure 3D). Finally, 16-month-old mice were tested in a novel place recognition paradigm. Similar to the novel object test, EC-hTau mice, and all control mice, showed a strong preference for spending time with an object that was in a new place compared to a familiar place (Figure 3E). There was also no difference between EC-hTau and NTG mice in time spent exploring the new place (two-sample t-test). With our group sizes, a 9.3% change between EC-hTau and NTG mice would have been enough to detect a significant effect (type I error rate of alpha = 0.05). Thus, taking into account the variety of behavioral tasks and the negative results from each, a conservative interpretation is that overexpression of mutant hTau in the entorhinal cortex and related structures is not sufficient to cause learning and memory deficits in a variety of tasks, at least given the time span, age range and strain background examined here.

### Age-dependent Expression of Pathological Tau in EC-hTau Mice

**Figure 4:**
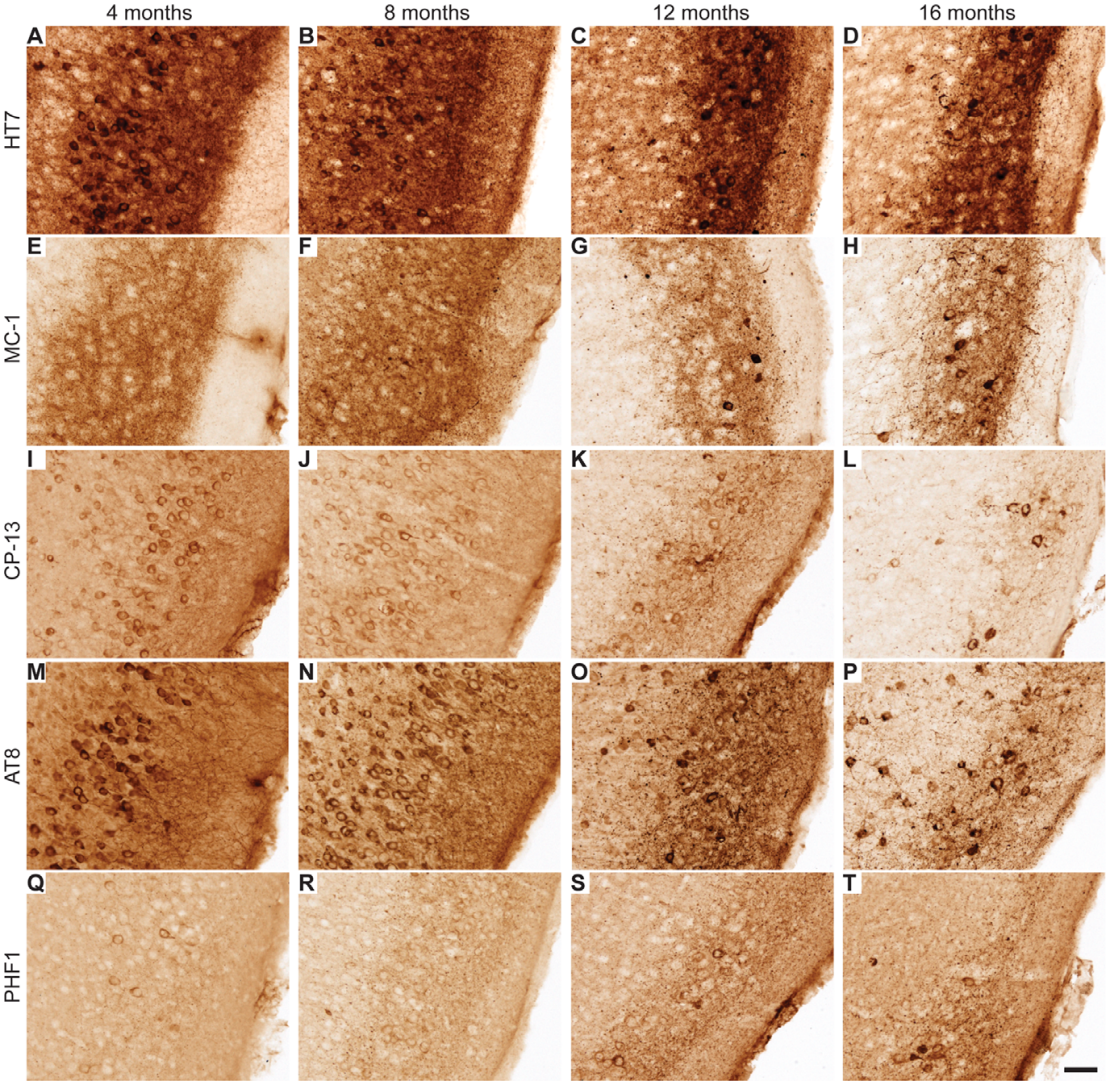
Expression of pathological tau in superficial EC layers of EC-hTau mice. (A–D) No obvious change in expression of the hTau transgene was observed between 4 and 16 months of age. (E–H) Abnormal conformation of tau, detected with the MC-1 antibody, was observed in EC neuropil at 4 months of age. At 12 and 16 months, cell bodies were also positively stained by MC-1 (G,H). (I–L) Phosphorylation of tau at serine 202 was detected with the CP-13 antibody. Cell bodies were positively stained for CP-13 across all ages, but some more intensely labeled neurons were observed at the older ages. CP-13 positive neuropil labeling also increased by 12 months (K). (M–P) Phosphorylations of tau at serine residues 199, 202, and 205 were detected with the AT8 antibody. Cell bodies were positively stained for AT8 across all ages. AT8 staining of the neuropil was maximal at 12 months (O). (Q–T) Phosphorylations of tau at serine 396 and 404 were detected with the PHF1 antibody. Scattered PHF1-positive cell bodies were seen at all ages. The neuropil and neurons were stained more intensely at 12 and 16 months (S,T). Scale bar (T) = 50 µm.

After behavioral testing, brains from the 16-month-old mice were examined for the presence and cellular localization of tau. In parallel, we analyzed 8-month-old mice that had been tested only once in the Morris water maze and cohorts of 4- and 12-month-old mice that had never been tested behaviorally. Total hTau, detected with the HT7 antibody, was most abundant in the superficial layers of the EC in EC-hTau mice (Figure 4A–D). Misfolded tau was detected with the MC-1 antibody [36]. In layers II/III of the EC, MC-1 staining was observed already in 4-month-old EC-hTau mice (Figure 4E). At 4 and 8 months, MC-1 stained primarily the neuropil (Figure 4E,F). By 12 months, MC-1 also labeled cell bodies (Figure 4G,H). Early phosphorylation of tau at serine 202 was detected with the CP-13 antibody in EC cell bodies of 4-month-old EC-hTau mice (Figure 4I). Cell bodies were CP-13 positive at all ages examined, but some neurons were more darkly stained with CP-13 at the older ages (Figure 4K,L). A similar pattern to CP-13 was observed after staining with the AT-8 antibody, which recognizes tau phosphorylated at serine residues 199, 202, and 205, although AT-8 densely labeled EC neuronal cell bodies even in the younger mice (Figure 4M–P). The PHF-1 antibody, which recognizes tau phosphorylated at serine residues 396 and 404 faintly stained neuronal cell bodies at 4 and 8 months (Figure 4Q, R) and densely labeled neuronal cell bodies and the neuropil at 12 and 16 months (Figure 4S, T). Thus, the EC of EC-hTau mice showed an age-dependent neuronal accumulation of misfolded and abnormally phosphorylated tau that is typically associated with AD and certain forms of FTLD. Based on the ages at which we first observed tau accumulation in neuronal cell bodies, abnormally phosphorylated tau detected by CP-13, AT-8 or PHF-1 appears to mislocalize to this compartment much earlier than misfolded tau detected by MC-1 (Figure 4).

**Figure 5:**
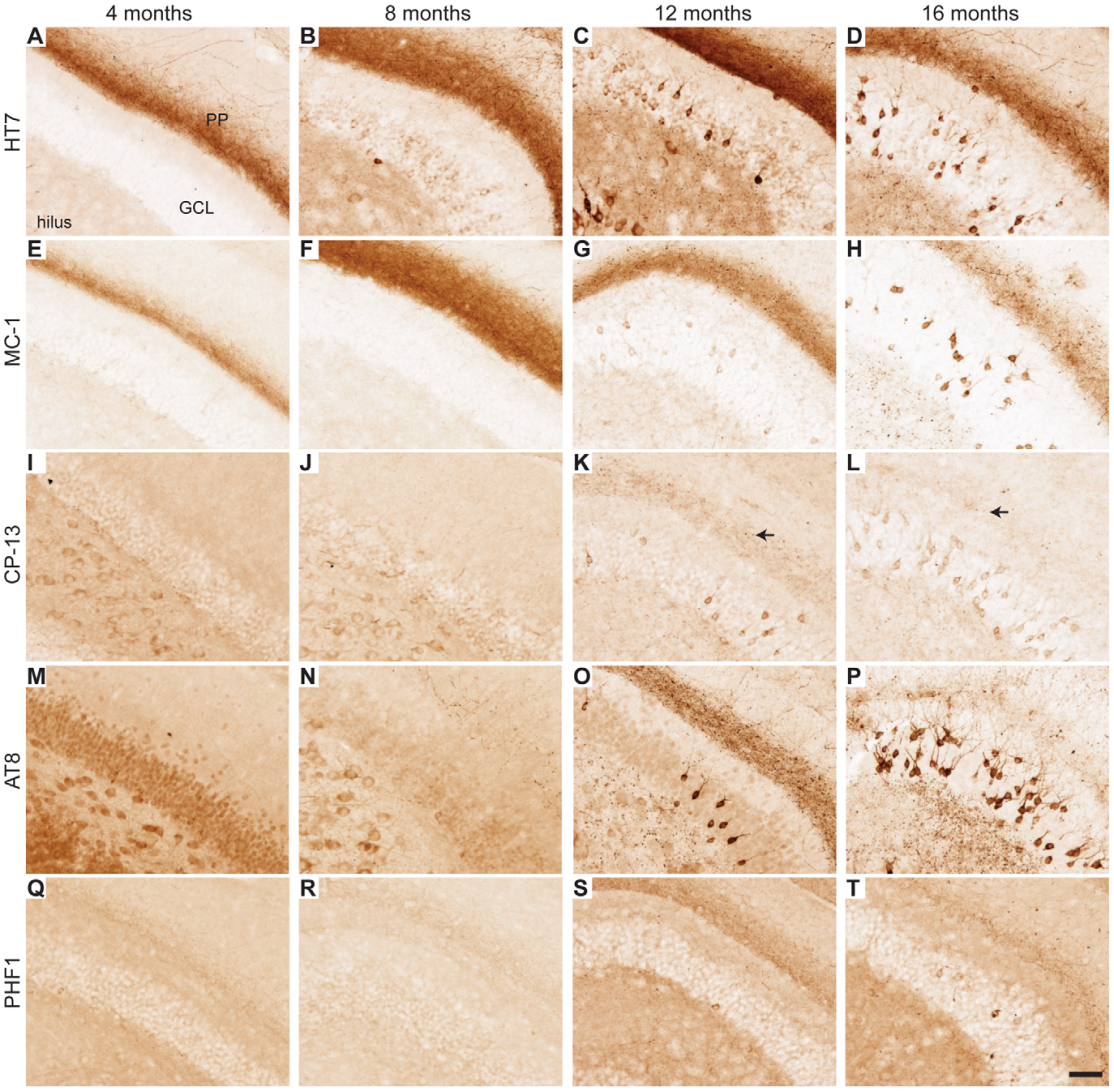
Pathological tau in the DG of EC-hTau mice. (A–D) hTau was detected in PP terminals in the outer molecular layer of the DG with the HT7 antibody at all ages examined. However, darkly stained cells in the granule cell layer (GCL) were seen primarily at 12 and 16 months (C, D). (E–H) PP terminals stained positive for MC-1 at all ages. By 12 and 16 months, GC also stained with MC-1 (G,H). (I–L) Faint CP13-positive PP terminals (arrows in K,L) and many more GC were observed at 12 and 16 months of age. (M–P) AT8 staining of the PP and GC increased markedly at 12 months (O) and many more positive GC were seen at 16 months (P). (Q–T) PHF1 staining of GC and the neuropil was also increased at 12 and 16 months (S,T). Scale bar (T) = 50 µm.

Tau pathology was also assessed in the hippocampus of EC-hTau mice. Dense total hTau expression was detected with the HT7 antibody in PP terminals in the molecular layer of the DG at all ages (Figure 5A–D). No hTau was detected in DG GC cell bodies of 4-month-old EC-hTau mice (Figure 5A). However, there was variable expression of hTau in mossy fibers at this age in ∼50% of mice (Figure S1). By 8 months, GC of EC-hTau mice were faintly, but consistently, labeled with the HT7 antibody (Figure 5B), and many GCs were intensely stained in 12- and 16-month-old mice (Figure 5C,D). Detection of various pathological forms of tau showed a similar age-dependent increase in staining intensities. Faintly MC-1-positive GCs were first observed in 12-month-old mice and prominent staining of cell bodies was observed at 16 months (Figure 5G,H). CP-13 and AT-8 also stained GCs above NTG levels in 12-month-old EC-hTau mice, and numbers of labeled GCs increased by 16 months (Figure 5I–P and Figure S2). PHF1 positive GCs were mostly observed in the 16-month age group (Figure 5T). We also observed a reduction in tau immunoreactivity of perforant path axons at the oldest age (compare 12 and 16 months in Figure 5). Data obtained by de Calignon et al. (2012) in a similar model suggest that this alteration may be a harbinger of axonal degeneration and subsequent neuronal loss in the EC.

**Figure 6:**
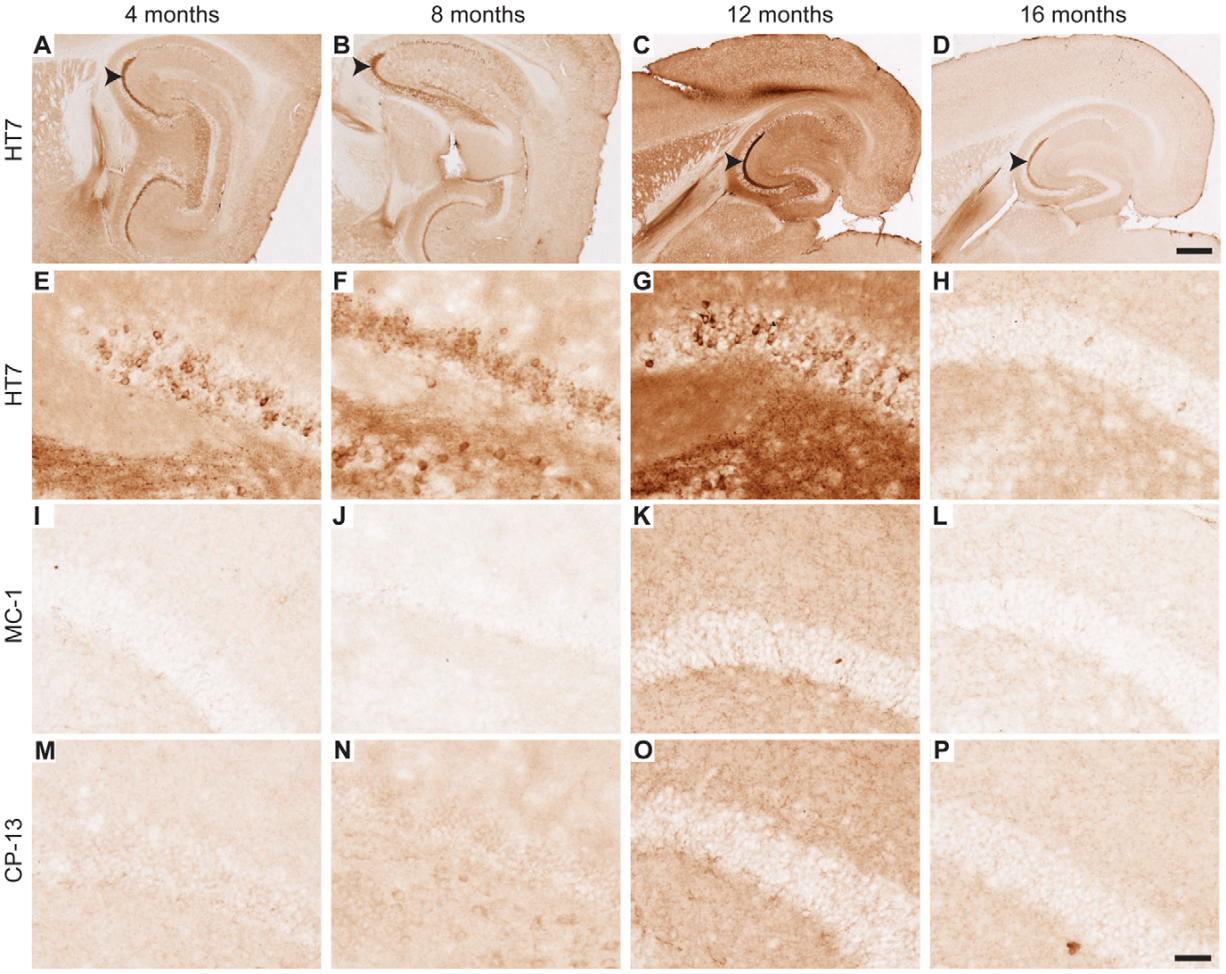
DG GC of tet-hTau singly transgenic mice express hTau but not abnormal tau. (A–P) Brain sections from tet-hTau singly transgenic mice of different ages were immunostained with the hTau-specific antibody HT7 (A–H) or with antibodies that recognize misfolded (MC1; I–L) or abnormally phosphorylated (CP-13; M–P) tau. (A–D) Low power images show prominent expression of hTau in GC axons (mossy fibers, arrowheads) at all ages. (E–H) Cell bodies of GC were also variably immunoreactive for hTau at all ages examined. (I–P) GC cell bodies of tet-hTau singly transgenic mice did not stain with MC-1 (I–L) or CP-13 (M–P). However, we did observe MC-1 positive MFs in tet-hTau singly transgenic mice at all ages (data not shown). Scale bars: 500 µm (A–D) and 50 µm (E–P).

Although tet-hTau singly transgenic mice showed no hTau accumulation in the EC (Figure 6A–D), they did have hTau in DG GCs at all ages examined (Figure 6E–H). hTau immunoreactivity was also detected in the mossy fiber axons of GC in both EC-hTau mice (Figure 1A–D) and tet-hTau singly transgenic mice (Figure 6A–D and Figure S1). Notwithstanding this transgene “leakiness” in tet-hTau singly transgenic mice, we could not detect any pathological forms of tau in GC bodies of these mice (Figure 6I–P).

Sections from control genotypes (Figures S2 and S3; tTA was indistinguishable from NTG and is not shown) were stained in parallel with sections from EC-hTau mice for all the antibodies against pathological forms of tau. On control sections, MC-1 and CP-13 resulted only in diffuse background staining. In contrast, AT8 faintly labeled cell bodies in the hippocampus and cortex and PHF1 faintly labeled axons in the mossy fiber pathway (arrows) and the outer molecular layer of the DG. However, this labeling of control sections could easily be distinguished from the intense immunoreactivities these antibodies showed on sections from EC-hTau mice (Figures 4–5).

**Figure 7:**
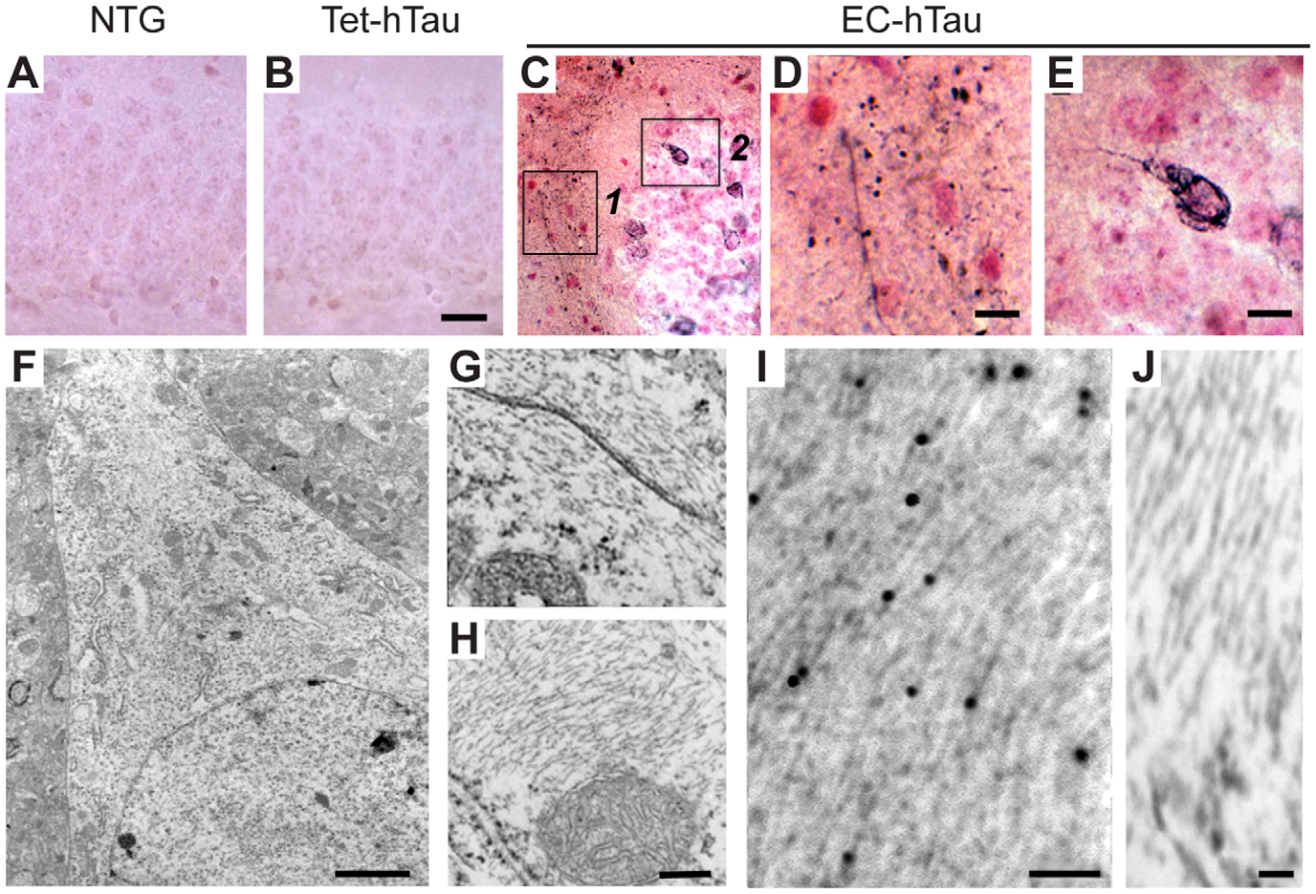
Tau aggregates in the DG of 16-month-old EC-hTau mice. (A–E) Gallyas silver staining revealed no abnormalities in NTG (A) and tet-hTau singly transgenic (B) controls. In contrast, EC-hTau mice had neuropil threads in the outer molecular layer of the DG (C, box 1 enlarged in panel D) and tangle-like inclusions in GC of the DG (box 2 enlarged in panel E). (F) Low magnification (5,000X) view of a GC. (G–H) Higher magnification (30,000X) view of intracellular filamentous aggregates in GC. (I) Immuno-EM analysis of the packed intracellular filaments with the PHF1 antibody. Gold particles decorate straight filaments; gold particles enhanced with silver solution are 15–20 nm. (J) Negative control (no primary antibody) shows the specificity of the immunogold labeling. Scale bars: 30 µm (A, B), 10 µm (D, E), 2 µm (F), 0.5 µm (G, H), 0.1 µm (I) and 0.05 µm (J).

At 16 months of age, EC-hTau mice, but none of the control groups, had neuropil threads in the outer molecular layer of the DG and GC inclusions reminiscent of tangles in AD patients, as demonstrated by Gallyas silver staining (Figure 7A–E and data not shown). Further analysis by electron microscopy revealed packed intracellular filamentous structures with an average diameter of 10–15 nm in GCs of EC-hTau mice (Figure 7F–I). Immuno-EM with the PHF1 antibody revealed many gold particles decorating these filaments (Figure 7I).

### Abnormal Tau Assemblies in the EC and DG of EC-hTau Mice

**Figure 8:**
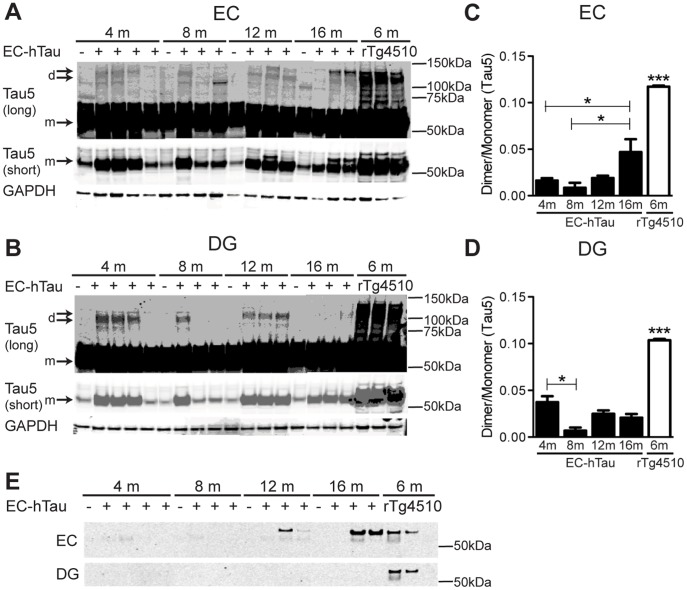
Biochemical detection of abnormal tau aggregation in EC-hTau mice. (A, B) Levels of tau dimers (d) in the EC (A) and DG (B) of EC-hTau mice, NTG mice (negative control) and rTg4510 mice (positive control) were detected by western blot analysis with antibodies against total tau (Tau5). The middle panels show shorter exposures of the tau monomer (m) band, used for optical density quantification. GAPDH was used as a loading control. (C–D) Ratios of tau dimers to monomers as determined by densitometric quantitation of western blot signals obtained from EC (C) and DG (D) homogenates. ***p<0.0005 vs. all EC-hTau groups or *p<0.05 as indicated by brackets (Tukey test). Values are mean ± SEM. (E) Sarcosyl-insoluble tau was extracted from the EC and DG of EC-hTau and rTg4510 mice and detected by western blot analysis with the Tau5 antibody.

Soluble tau dimers have been implicated in the pathogenesis of cognitive deficits [30]. Western blot analysis of brain tissues subdissected from EC-hTau mice of different ages revealed roughly 3-fold higher expression of hTau compared to endogenous mouse tau (data not shown), and variable levels of tau dimers in the EC and DG (Figure 8A, B). Quantitation of tau dimer-to-monomer ratios revealed significant age-dependent increases in the EC by 16 months of age, but not in the DG (Figure 8C, D). In both brain regions, dimer levels were much lower in EC-Tau mice than in rTg4510 mice, in which the same tet-hTau transgene is directed by a CaMKII-tTA driver line [6]. Variable levels of sarcosyl-insoluble tau were detected in the EC, but not the DG, of EC-hTau mice at 12 and 16 months of age (Figure 8E). In contrast to 6-month-old rTg4510 mice, 4- and 8-month-old EC-hTau mice had no detectable insoluble tau.

### Abnormalities of PP to Granule Cell Synapses in EC-hTau Mice

**Figure 9:**
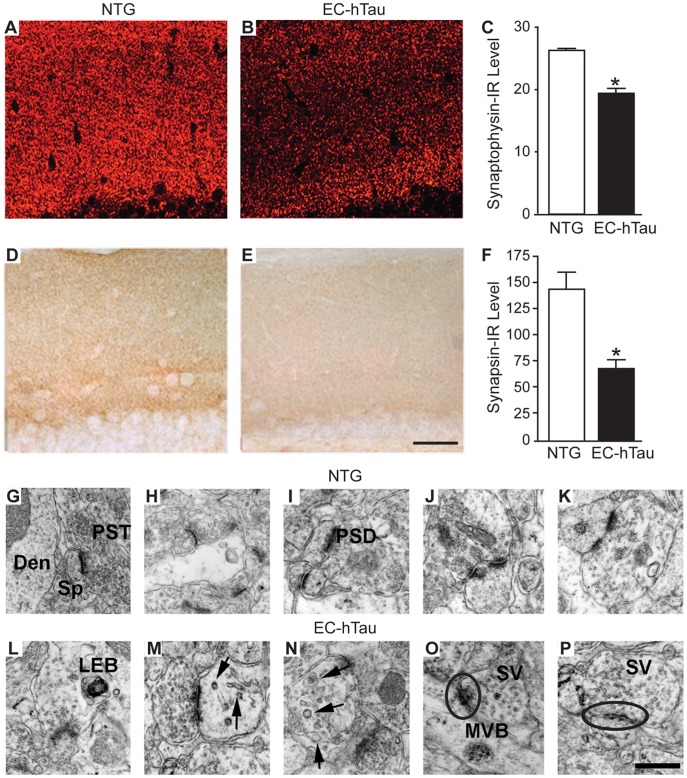
Synaptic alterations in the DG of 16-month-old EC-hTau mice. (A–F) Sagittal brain sections of NTG and EC-hTau mice were immunostained for synaptophysin (A–C) or synapsin (D–F). Levels of immunoreactive terminals were quantitated in the DG molecular layer (C, F). n = 8 mice per group. *p<0.05 (Student’s t test). Values are mean ± SEM. (G–P) Ultrastructural analysis of synaptic alterations in EC-hTau mice. Electron micrographs were obtained at 25,000X from the molecular layer of the DG. Representative images from NTG controls (G–K) show presynaptic terminals (PST), spines (Sp) and dendrites (Den) with normal characteristics, including abundant round, clear synaptic vesicles and dense postsynaptic apparatus. In EC-hTau mice (L–P), presynaptic terminals were enlarged and irregular with laminated electrondense bodies (LEB), vesicular-tubular structures (arrows in M), enlarged vesicles (arrows in N), a paucity of small synaptic vesicles (SV), and a diffuse appearance of postsynaptic sites (encircled). Dendrites of EC-hTau mice also contained multivesicular bodies (MVB in O). Scale bars = 20 µm (in E for A, B, D and E) and 0.5 µm (in P for G–P).

Synaptic loss in the hippocampus correlates well with memory deficits in AD patients [37], [38], and APP transgenic mouse models also show synaptic deficits [39]–[42]. Although 16-month-old EC-hTau mice had no detectable deficits in hippocampus-dependent learning and memory (see above), they had significant losses of the presynaptic markers synaptophysin (Figure 9A–C) and synapsin (Figure 9D–F) in the outer molecular layer of the DG. They also showed reductions in the dendritic spine marker spinophilin in this layer, as well as a trend toward reduced levels of the postsynaptic density protein PSD-95 (data not shown). In addition to depletion of pre- and postsynaptic proteins, EC-hTau mice had ultrastructural abnormalities at PP to GC synapses in the molecular layer of the DG (Figure 9G–P). Their presynaptic terminals contained laminated electrondense bodies and vesicular-tubular structures and had enlarged vesicles as well as fewer small synaptic vesicles compared to NTG controls. Changes in postsynaptic specializations of EC-hTau mice consisted primarily of diffuse-appearing postsynaptic densities and multivesicular bodies in GC dendrites.

**Figure 10:**
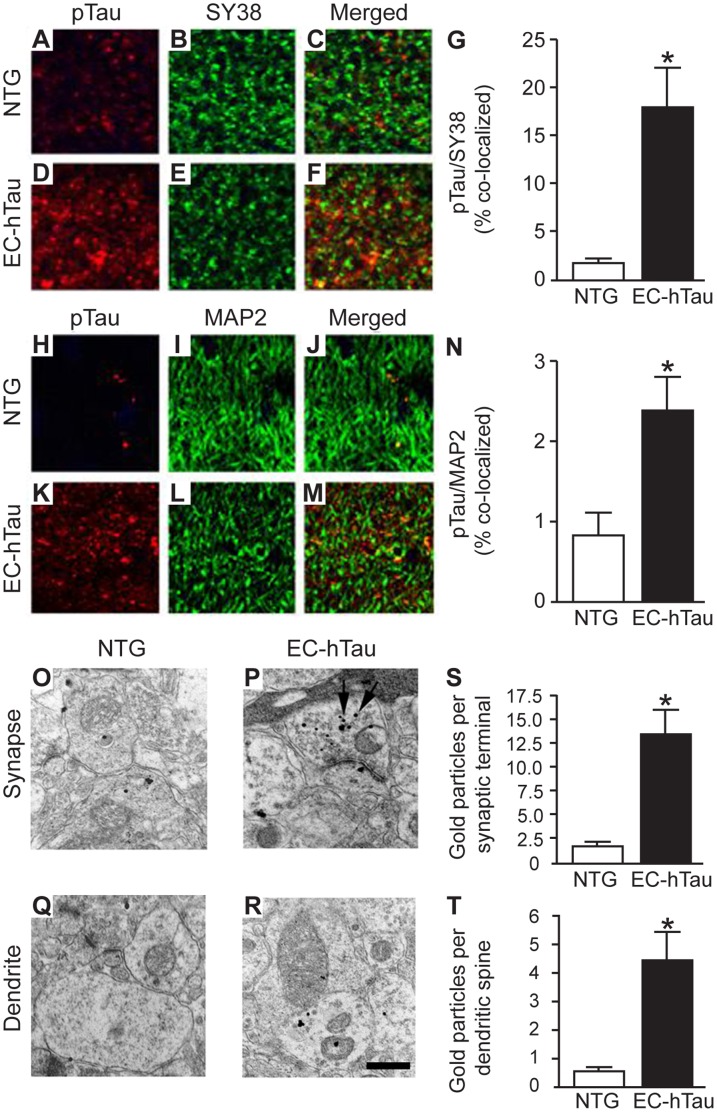
Enrichment of PHF1-tau in PP to GC synapses of 16-month-old EC-hTau mice. (A–F) Images from the outer molecular layer of the DG from sections of EC-hTau and NTG mice colabeled for pTau (PHF1, red) and synaptophysin (SY38, green). (G) Quantification of the number of synaptophysin-positive punctae that were also positive for pTau. (H–M) Images from the outer molecular layer of the DG from sections of EC-hTau and NTG mice colabeled for pTau (PHF1, red) and MAP2 (green). (N) Quantification of the number of MAP2-positive structures that were also positive for pTau. (O–R) Immuno-EM images of PP to GC synapses in the molecular layer of the DG from immunogold (PHF1)-labeled sections of NTG and EC-hTau mice. Arrows in (P) indicate gold particles. (S, T) Quantification of gold particles in presynaptic (S) and postsynaptic (T) structures. n = 8 mice per group, *p<0.05 (Student’s t test). Values are mean ± SEM.

To define the localization of phosporylated tau (pTau) at PP to GC synapses, sections from EC-hTau and NTG mice were double-labeled with PHF1 and antibodies to synaptophysin (SY38; Figure 10A–F) or MAP2 (Figure 10H–M). A greater proportion of presynaptic terminals (Figure 10G) and dendrites (Figure 10N) was colabeled with PHF1 in EC-hTau than in NTG mice. Immuno-EM with PHF1 revealed more gold particles in presynaptic terminals and dendritic spines in EC-hTau mice than in NTG controls (Figure 10O–T).

## Discussion

Our study demonstrates that overexpression of P301L-mutant hTau in the EC is insufficient to cause cognitive deficits in mice up to 16 months of age, even though it causes extensive hyperphosphorylation and abnormal folding of tau as well as tau aggregation and synaptic abnormalities. These results contrast with our recent findings that overexpression of mutant APP/Aβ in the EC causes not only synaptic deficits, but also age-dependent cognitive and behavioral abnormalities [23]. Taken together, these studies suggest a predominant role of APP/Aβ in the pathogenesis of early neuronal dysfunction in the entorhinal-hippocampal network. They do not exclude critical roles of tau in the pathogenesis of AD-related dysfunction of other brain regions or in the loss of neurons that occurs as the disease progresses in humans. Evidence for such roles of tau has been obtained in multiple lines of transgenic mice with widespread neuronal expression of hTau and may involve disruptions of axonal transport, destabilization of microtubules, mislocalization of tau into dendritic spines, and changes in neurotransmission [43], [44]. It should also be noted that hAPP transgenic mice that lack endogenous tau are protected from developing many of the synaptic, network and cognitive alterations seen in hAPP mice with wildtype tau levels [45]–[49], suggesting a critical role of endogenous wildtype tau in Aβ-induced neuronal dysfunction.

Although no tau mutations have been discovered in AD patients, many transgenic lines of mice with neuronal overexpression of FTLD-mutant or wildtype forms of hTau simulate typical AD pathologies, including hyperphosphorylation, aggregation and missorting of tau, and neuronal loss in vulnerable brain regions [43], [44]. Cognitive deficits have also been reported in tau transgenic mice with widespread transgene expression [6], [24], [32]–[34], [50]. However, since these transgenic mice overexpress hTau throughout most of the brain, it is difficult to know whether the resulting cognitive deficits are due specifically to tau-induced dysfunction of AD-vulnerable brain regions (e.g. entorhinal-hippocampal areas), as opposed to brain regions vulnerable to other tauopathies with cognitive symptoms (e.g. frontal cortex).

In contrast to these models, EC-hTau mice at ages up to 16 months were not cognitively impaired relative to their littermate controls. We calculated statistical power curves to ensure that the lack of differences between EC-hTau mice and control groups in our behavioral tests was not spurious. As reported in the Results section, our current sample sizes and variances were such that we could have detected statistically significant learning deficits in EC-hTau mice if their average latencies in the Morris water maze had increased by 6.2–9.4 sec over average latencies found in control groups. Such increases are smaller or approximately equal to those found in transgenic lines with widespread hAPP expression [26], [35] and with EC-restricted expression of mutant APP [23], supporting the conclusion that the absence of detectable behavioral deficits in the EC-hTau line is real. However, more subtle deficits could still have been missed.

It is important to consider in this regard whether repeated exposure and training in the same behavioral tests might have helped EC-hTau mice overcome subtle deficits. However, behaviorally naïve 8-month-old EC-hTau mice were also unimpaired in the Morris water maze. Furthermore, EC-hTau mice were tested for the first time in the passive avoidance paradigm at 12 months and in the novel place recognition paradigm at 16 months and showed no deficits in either paradigm as compared to age-matched NTG controls. Lastly, because the levels of tau dimers and insoluble tau in the EC and DG were clearly lower in EC-hTau mice than in rTg4510 mice, it is possible that the levels of functionally relevant abnormal tau assemblies were simply not high enough in EC-hTau mice to cause significant behavioral impairments.

The EC and its connections to the hippocampus have an established role in spatial navigation memory [25], [51], [52]. We were therefore surprised that EC-hTau mice with overt tau pathology within the EC and pathological alterations of EC to GC synapses did not display any measurable cognitive deficits, particularly since the same paradigms revealed obvious deficits in transgenic mice expressing APP/Aβ in a similar distribution [23]. The EC-hTau mice tested here were F1 hybrids between C57Bl6 and FVBN strains instead of congenic C57Bl6 as our EC-APP mice [23] and, thus, might have benefited from hybrid vigor. However, hAPPJ20 mice on the same FVBN/C57Bl6 background did display cognitive deficits relative to their NTG littermates [53]. Interestingly, virus-mediated overexpression of wildtype hTau produced tau pathology within the hippocampus but was also not sufficient to cause cognitive impairment [54]. Extensive aggregation of tau in both EC and hippocampal regions may be required to cause cognitive decline [55].

Whereas several transgenic lines with widespread neuronal expression of P301L-mutant hTau develop cognitive deficits [6], [24], [56], others do not [50], or have improved cognition at younger ages before developing deficits at old ages [57], [58]. The P301L-mutant hTau line reported by Kimura et al. (2007) had no cognitive deficits but extensive tau pathology and neuronal loss, whereas a complementary line expressing wildtype hTau had cognitive deficits but less tau pathology and no neuronal loss [50]. Phosphorylation of wildtype tau in the EC of these mice correlated with synaptic loss, impairment of neuronal activity, and cognitive deficits [50]. Thus, it is possible that wildtype, but not P301L-mutant, hTau can cause neuronal dysfunction of the EC.

At first glance, the EC-hTau model may seem suitable for testing recent hypotheses on “prion-like” spread of tau pathology between cells and interconnected brain regions [18], [19], [59]–[61], because tTA in the neuropsin-tTA line is expressed in presynaptic EC cells but not in postsynaptic DG GCs [25] and this expression pattern was confirmed in EC-APP mice [23]. However, the tet-hTau singly transgenic line has some level of “leaky” transgene expression in the absence of the transactivator, including in GCs of the DG ([62] and this study). Consequently, hTau is weakly expressed in the DG of EC-hTau mice, albeit at much lower levels than in the EC. These findings are consistent with those reported by others in similar EC-hTau mice [63], [64]. In our opinion, the expression of hTau in GCs of tet-hTau mice makes it impossible to definitely conclude that hTau was transferred from EC into DG neurons in the EC-hTau model. Using a similar model, another group recently showed that GCs containing hTau protein were devoid of hTau mRNA [64] and concluded that hTau protein must have been transferred into these cells from other neurons. Another possible interpretation is that high levels of pathological hTau protein in GCs caused a reduction in mRNA synthesis, as has been reported for tangle-bearing neurons [65].

Notwithstanding these caveats, we did find pathological forms of tau in GC bodies in EC-hTau mice, as also observed by [63], [64], but not in tet-hTau singly transgenic mice. Therefore, the “leaky” expression of hTau in GCs cannot account for the accumulation of pathological tau in GCs of EC-hTau mice. These findings raise two possibilities. First, pathological tau may have been transferred from EC to DG neurons, causing the accumulation of pathological tau within GCs, possibly enabled or promoted by low levels of corresponding hTau “templates” in GCs (indicating a prion-like behavior). In support of this hypothesis, hTau was found to co-aggregate with endogenous mouse tau [64]. However, in P301L FTD patients, mutant tau actually does not seem to sequester wildtype tau [66], possibly because “seeds” of P301L tau induce the assembly of tau filaments from P301L-mutant, but not wildtype, tau [67]. Second, overexpression and accumulation of tau in the EC may have indirectly caused the abnormal localization and conformational changes of hTau expressed by GCs, perhaps through alterations in network activity and afferent inputs. Additional studies are needed to distinguish between these possibilities.

The transgenic tau model presented here recapitulates both the topological pattern of tau pathology and the lack of cognitive deficits in AD patients with early Braak stages. It could be utilized to investigate how additional factors such as Aβ, apolipoprotein E4, α-synuclein or TDP-43, may advance the progression of AD beyond these early stages. A better understanding of this transition could provide additional avenues for therapeutic intervention to prevent loss of memory and other cognitive functions.

## Supporting Information

Figure S1 **Total hTau antibody staining of brain sections from 4–5-month-old tet-hTau, EC-hTau and NTG mice.** Brains were cut in the sagittal (A–K, M–O, Q–R) or horizontal (L, P) plane and sections were immunostained with the hTau-specific polyclonal antibody E1. No hTau was detected in the EC of tet-hTau (A–F) and NTG (M–R) mice. “Leaky” hippocampal expression of the hTau transgene was observed in some tet-hTau mice but not others: the mossy fibers (MF, arrowheads) of DG granule cells stained positively for hTau in four of six singly transgenic tet-hTau mice (A–F). Mossy fibers were also stained for hTau in three of six EC-hTau mice (G–L). Only diffuse background staining was observed in NTG mice (M–R). Scale bar: 500 µm.
(PNG)

Figure S2 **Brain sections from NTG mice stained with antibodies to pathological forms of tau.** Brains from NTG mice of the indicated ages were cut in the sagittal (4 and 8 months) or horizontal (12 and 16 months) plane and sections were immunostained with MC-1, CP-13, AT8, and PHF1, in parallel with sections from age-matched tet-hTau (Figure S3, Figure 6), tTA (data not shown), and EC-hTau mice (Figures 4, 5, 6). MC-1 and CP-13 resulted mainly in diffuse background staining (top rows). The AT8 antibody, which detects serine phosphorylation of tau at residues 199, 202, and 205, faintly labeled cell bodies (arrowheads) in the hippocampus and cortex at all ages examined. PHF1, which detects tau phosphorylated at serines 396 and 404, faintly labeled axons in the mossy fiber pathway (arrows) and in the outer molecular layer of the DG. Scale bar: 500 µm.
(PNG)

Figure S3 **Brain sections from tet-hTau mice stained with antibodies to pathological forms of tau.** Brains from tet-hTau mice of the indicated ages were cut in the sagittal (4 and 8 months) or horizontal (12 and 16 months) plane and sections were immunostained with MC-1, CP-13, AT8, and PHF1, in parallel with sections from age-matched NTG (Figure S2), tTA (data not shown), and EC-hTau mice (Figures 4, 5, 6). The AT8 antibody faintly labeled cell bodies (arrowheads) in the hippocampus and cortex, and the PHF1 antibody faintly labeled axons in the mossy fiber pathway (arrows) and the outer molecular layer of the DG. The distribution and intensity of AT8 and PHF1 immunoreactivities were similar to those observed in NTG controls (Fig. S2). Scale bar: 500 µm.
(PNG)
