# Supplementary figures and images for: Human P301L-Mutant Tau Expression in Mouse Entorhinal-Hippocampal Network Causes Tau Aggregation and Presynaptic Pathology but No Cognitive Deficits
Source: PLoS One. 2012 Sep 24;7(9):e45881. doi: 10.1371/journal.pone.0045881 (PMC3454317; doi:10.1371/journal.pone.0045881)

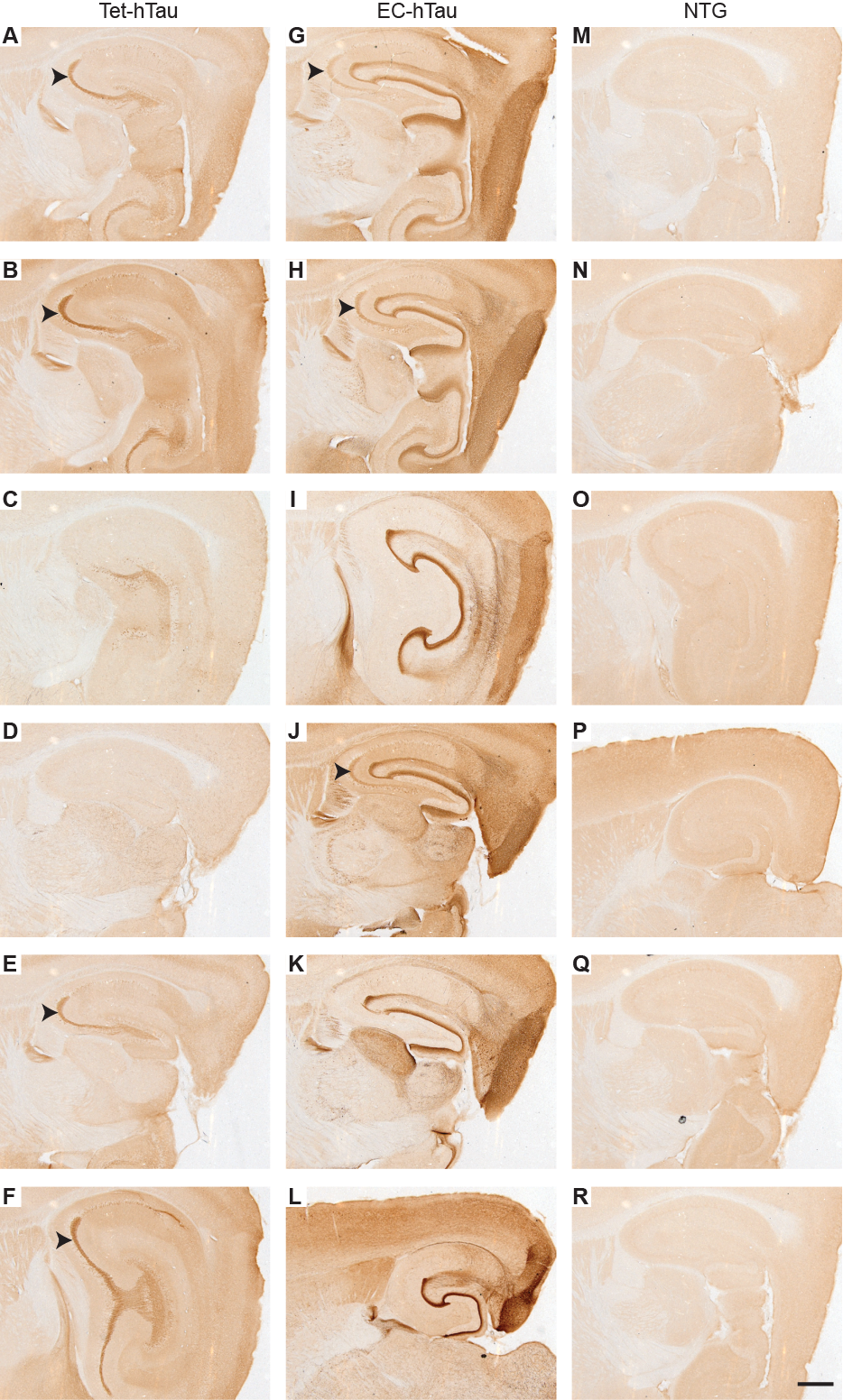

Supplement: Figure S1 — Total hTau antibody staining of brain sections from 4–5-month-old tet-hTau, EC-hTau and NTG mice. Brains were cut in the sagittal (A–K, M–O, Q–R) or horizontal (L, P) plane and sections were immunostained with the hTau-specific polyclonal antibody E1. No hTau was detected in the EC of tet-hTau (A–F) and NTG (M–R) mice. “Leaky” hippocampal expression of the hTau transgene was observed in some tet-hTau mice but not others: the mossy fibers (MF, arrowheads) of DG granule cells stained positively for hTau in four of six singly transgenic tet-hTau mice (A–F). Mossy fibers were also stained for hTau in three of six EC-hTau mice (G–L). Only diffuse background staining was observed in NTG mice (M–R). Scale bar: 500 µm. (PNG) [file pone.0045881.s001.png]

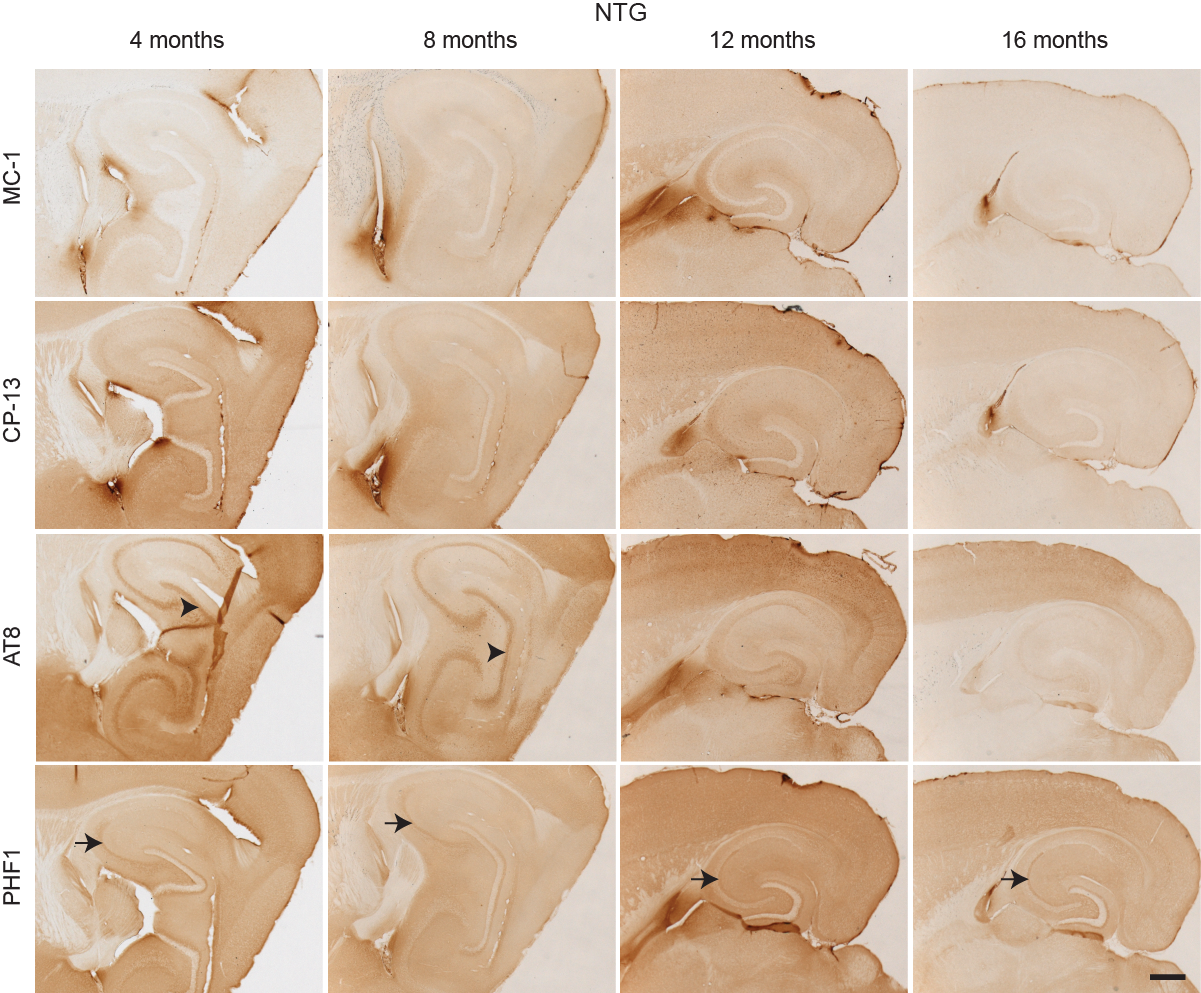

Supplement: Figure S2 — Brain sections from NTG mice stained with antibodies to pathological forms of tau. Brains from NTG mice of the indicated ages were cut in the sagittal (4 and 8 months) or horizontal (12 and 16 months) plane and sections were immunostained with MC-1, CP-13, AT8, and PHF1, in parallel with sections from age-matched tet-hTau (Figure S3, Figure 6), tTA (data not shown), and EC-hTau mice (Figures 4, 5, 6). MC-1 and CP-13 resulted mainly in diffuse background staining (top rows). The AT8 antibody, which detects serine phosphorylation of tau at residues 199, 202, and 205, faintly labeled cell bodies (arrowheads) in the hippocampus and cortex at all ages examined. PHF1, which detects tau phosphorylated at serines 396 and 404, faintly labeled axons in the mossy fiber pathway (arrows) and in the outer molecular layer of the DG. Scale bar: 500 µm. (PNG) [file pone.0045881.s002.png]

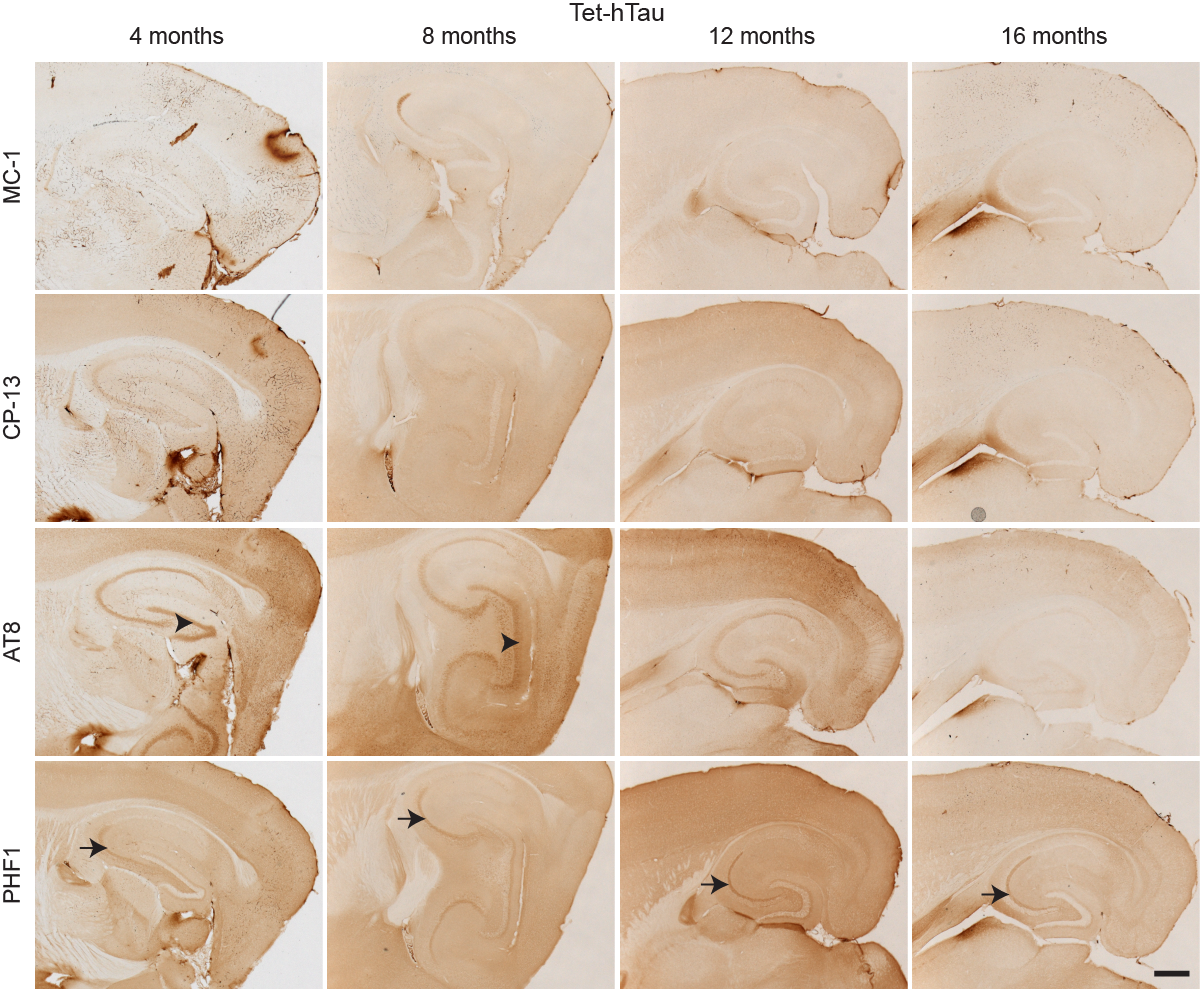

Supplement: Figure S3 — Brain sections from tet-hTau mice stained with antibodies to pathological forms of tau. Brains from tet-hTau mice of the indicated ages were cut in the sagittal (4 and 8 months) or horizontal (12 and 16 months) plane and sections were immunostained with MC-1, CP-13, AT8, and PHF1, in parallel with sections from age-matched NTG (Figure S2), tTA (data not shown), and EC-hTau mice (Figures 4, 5, 6). The AT8 antibody faintly labeled cell bodies (arrowheads) in the hippocampus and cortex, and the PHF1 antibody faintly labeled axons in the mossy fiber pathway (arrows) and the outer molecular layer of the DG. The distribution and intensity of AT8 and PHF1 immunoreactivities were similar to those observed in NTG controls (Fig. S2). Scale bar: 500 µm. (PNG) [file pone.0045881.s003.png]
